# A novel approach to the selection of an appropriate pacing position for optimal cardiac resynchronization therapy using CT coronary venography and myocardial perfusion imaging: FIVE STaR method (fusion image using CT coronary venography and perfusion SPECT applied for cardiac resynchronization therapy)

**DOI:** 10.1007/s12350-019-01856-z

**Published:** 2019-08-21

**Authors:** Tomohiro Tada, Koichi Osuda, Tomoaki Nakata, Ippei Muranaka, Masafumi Himeno, Shingo Muratsubaki, Hiromichi Murase, Kenji Sato, Masanori Hirose, Takayuki Fukuma

**Affiliations:** 1 Department of Cardiology, Hakodate Goryoukaku Hospital, 38-3 Goryoukaku, Hakodate, Hokkaido 040-8611 Japan; 2 Division of Clinical Radiology Services, Hakodate Goryoukaku Hospital, 38-3 Goryoukaku, Hakodate, Hokkaido 040-8611 Japan

**Keywords:** CT, SPECT, Heart failure

## Abstract

**Background:**

Nearly one-third of patients with advanced heart failure (HF) do not benefit from cardiac resynchronization therapy (CRT). We developed a novel approach for optimizing CRT via a simultaneous assessment of the myocardial viability and an appropriate lead position using a fusion technique with CT coronary venography and myocardial perfusion imaging.

**Methods and Results:**

The myocardial viability and coronary venous anatomy were evaluated by resting Tc-99m-tetrofosmin myocardial perfusion imaging (MPI) and contrast CT venography, respectively. Using fusion images reconstructed by MPI and CT coronary venography, the pacing site and lead length were determined for appropriate CRT device implantations in 4 HF patients. The efficacy of this method was estimated by the symptomatic and echocardiographic functional parameters. In all patients, fusion images using MPI and CT coronary venograms were successfully reconstructed without any misregistration and contributed to an effective CRT. Before the surgery, this method enabled the operators to precisely identify the optimal indwelling site, which exhibited myocardial viability and had a lead length necessary for an appropriate device implantation.

**Conclusions:**

The fusion image technique using myocardial perfusion imaging and CT coronary venography is clinically feasible and promising for CRT optimization and enhancing the patient safety in patients with advanced HF.

**Electronic supplementary material:**

The online version of this article (10.1007/s12350-019-01856-z) contains supplementary material, which is available to authorized users.

## Introduction

Because of the increase in the aging population and medical cost, an appropriate management of patients with refractory or advanced heart failure (HF) has become one of the important clinical issues to be resolved in the developed countries.^1^-^4^ In addition to the advancements in drug treatment, device implantations using implantable cardioverter-defibrillators and/or cardiac resynchronization therapy (CRT) devices have contributed to the improvement in the outcomes in patients with advanced HF.^5^-^7^ Roughly one-third of patients undergoing CRT, however, does not benefit from this device treatment,^7^-^10^ even though the indication criteria for CRT have regularly been revised.^11^-^13^ There are several proposed mechanisms behind a non-response to CRT. Overall or non-cardiac conditions can be involved in the poor outcomes in HF patients undergoing CRT. Current CRT criteria are limited in their ability to select appropriate candidates, particularly for the assessment of the grade and volume of electrical and/or mechanical dyssynchrony in the left ventricle and underlying myocardial injury in those that cannot respond to CRT. Delgado et al demonstrated that myocardial scar in the region of the LV pacing lead is an independent determinant of the long-term prognosis in ischemic HF.^14^ Technical approaches for device implantations are also important for achieving effective pacing for synchronization of the cardiac contraction. Functional improvement during systole with CRT is more likely to depend on the mechanical dyssynchrony of the entire left ventricle than a narrow range of the electrical dyssynchrony or single-sectional assessment by a 2D echo study.^9^,^10^

Under this context, we realized that both the assessment of the myocardial viability and appropriate selection of the pacing site in viable myocardium were indispensable for a sufficient responsiveness to CRT and prognostic improvement. Recently we developed a novel approach, named the *FIVE STaR* method (Fusion Image using CT Venography and perfusion SPECT applied for cardiac Resynchronization therapy), to optimize the efficacy of the CRT by discriminating CRT responders from non-responders. This method is a three-dimensional fusion technique that simply combines the two images derived from the contrast computed tomography (CT) coronary venography and gated myocardial perfusion SPECT imaging. Myocardial perfusion imaging is widely used for the assessment of inducible ischemia, myocardial viability, and cardiac event risks in patients with known or suspected coronary artery disease. On the other hand, the recent improvement in the temporal and spatial resolutions has enabled multi-slice cardiac CT imaging to clearly depict the coronary anatomy, in particular coronary vessels and coronary atherosclerotic lesions.

In this study, we applied a three-dimensional fusion imaging technique using the *FIVE STaR* method for the delineation of both the coronary venous system and myocardial viability via the perfusion tracer activity. This study aimed to evaluate the clinical feasibility and efficacy of this method for optimizing the CRT via a myocardial viability assessment and appropriate selection of the pacing site in patients with advanced HF.

## Methods

Based on the guidelines for device treatment currently available^11^-^13^ and the ethical committee guidelines in our hospital, four consecutive patients were selected as CRT candidates at the heart team conference and enrolled in this study after obtaining informed consent. A defibrillation function was added in three patients using a cardiac resynchronization therapy and defibrillator (CRTD) device.

### Myocardial Perfusion SPECT Imaging

At rest, myocardial perfusion SPECT imaging was performed 30 min after an intravenous injection of Tc-99m-labeled Tetrofosmin (296 MBq) in an electrocardiogram-gated (ECG) mode. A single-head gamma camera equipped with a high-resolution, parallel-hole collimator (Infinia, GE Healthcare Japan) was used to collect data at 180° (6° × 30) using the Step & Shoot method. Short-axis SPECT images were reconstructed and depicted as a polar map using serial short-axis images together with a display of the regional percent uptake (%) of the tracer activity, then a three-dimensional image was reconstructed for the following fusion imaging (Xeleris, GE Healthcare Japan).

### Contrast CT Venography

CT coronary venography was performed using a 320-detector-row multi-slice CT (Aquilion ONE, Canon Medical Systems) with an image processing system (ziostation2, Ziosoft). The patients underwent an intravenous injection of contrast agent (190 mgI/kg) and beta-blockers if necessary, to keep the heart rate at less than 60 bpm. CT imaging with an ECG-gating mode was started at the time when the coronary veins were heavily stained following the Test Bolus Tracking method to reduce the radiation exposure and amount of contrast agent. In order to avoid any image distortion, motion artifact, and misregistration, the cross sections obtained during the same phase were used for the three-dimensional reconstruction and subsequent image fusion with the perfusion SPECT data. The CT images were reconstructed for each part of the left ventricle, coronary veins, right atrium, tricuspid valve, right ventricle, pulmonary artery, and spine. Then all images were integrated into one three-dimensional image.

For the appropriate placement of the pacing lead, a path was drawn along the targeted coronary vein to the suggested pacing site with viable myocardium identified by where the perfusion SPECT image overlapped. This method enabled the operators to precisely measure the pacing lead length and to three-dimensionally determine the appropriate pacing site. This method also contributed to the three-dimensional identification of the tricuspid annulus and lumbar spine, their correlations to the coronary veins on the same screen, and to the selection of an appropriate projection view for the device replacement.

### CRT Device Implantation

The CRT device implantation was performed by monitoring the CT/SPECT fusion image by referring to the surrounding organs such as the vertebral canal and tricuspid valve as follows. A guiding catheter was inserted to the appropriate site in the targeted coronary vein as selected in advance, then the pacing lead was positioned using a subselection catheter (if necessary). The wire was advanced to the distal part of the coronary vein by the over-the-wire method and the lead was successfully introduced. The post-operative efficacy of the CRT was evaluated by a decrease in the left ventricular end-systolic volume (LVESV, mL), and an increase in the left ventricular ejection fraction (LVEF, %) or New York Heart Association (NYHA) functional class. The definition of a CRT responder was as follows: a 15% or more decrease in the LVESV, 5% or more increase in the LVEF, and one grade or more increase in the NYHA class at 3 months or later.

## Results

In all patients, three-dimensional fusion images of the gated SPECT images and CT coronary venograms were successfully reconstructed without any definitive misregistration. Before the implantation, the fusion images enabled the operators to plan the appropriate approach to an optimal indwelling site among the coronary vein tree in which the myocardial viability was preserved with a % tracer uptake of 50% or more. The appropriate coronary vein and pacing point were selected from the CT venogram overlapped with the myocardial perfusion image, and then the appropriate curve shape and precise length of the pacing lead were successfully measured for an appropriate device implantation in all patients.

**Figure 1:**
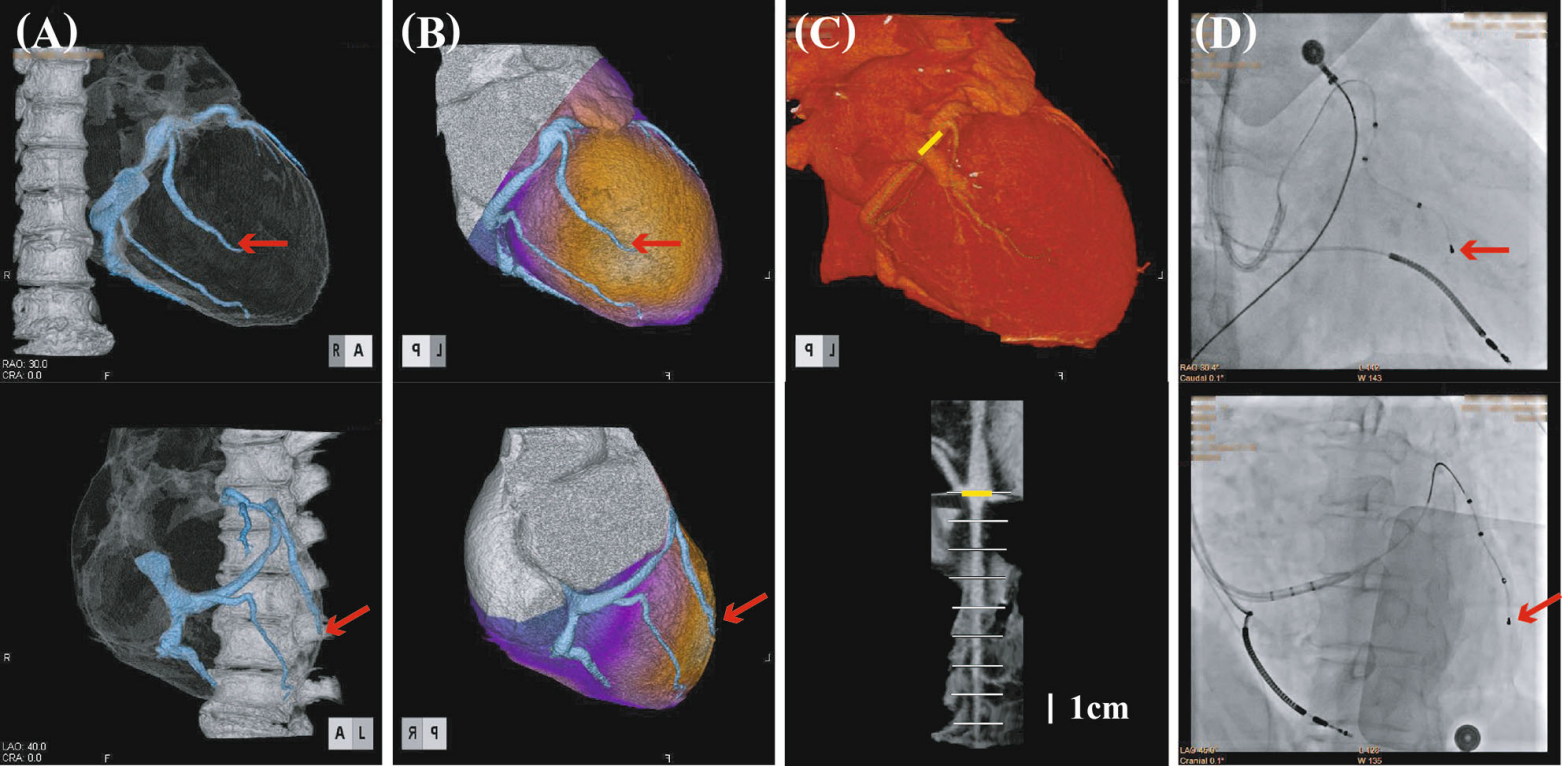
Case 1: A 60-year-old female with idiopathic dilated cardiomyopathy. The vertebral canal and coronary CT venograms are clearly reconstructed (**A**) then the coronary perfusion image is overlapped (**B**). The target coronary vein (*red arrows*) is easily selected as an appropriate pacing site in viable myocardium (*yellow areas in B*). Following the measurement of the vein length (**C**), the CRT pacing lead is appropriately located as planned in advance (*black arrows*, **D**). Two months later, the end-systolic volume decreased from 188 to 133 mL (a 27% reduction), left ventricular ejection fraction increased from 26 to 31%, and NYHA functional class improved from class III to class II

**Figure 2:**
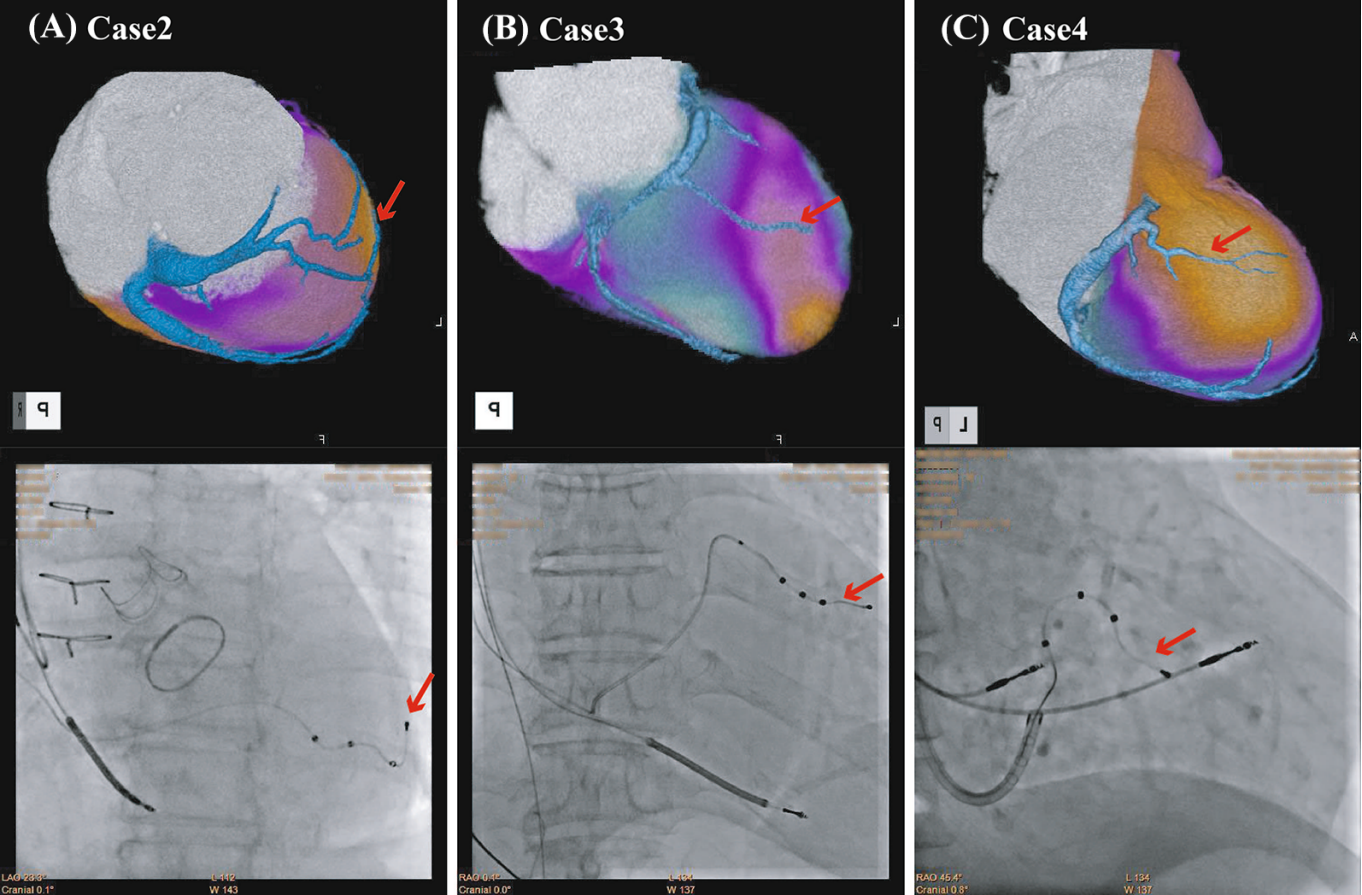
The fusion images of the myocardial perfusion image and coronary CT venogram (*upper panels*) in 3 cases. In all cases, the CRT pacing leads are appropriately located at viable myocardium (*lower panels)*. (**A**) Case 2, a 70-year-old female with idiopathic dilated cardiomyopathy (DCM) and a post-aortic valve replacement; (**B**) Case 3, a 60-year-old male with cardiac sarcoidosis; (**C**) Case 4, a 90-year-old male with DCM

Table 1 summarizes the post-operative alterations in the LVESV, LVEF, and NYHA functional class in the 4 patients (Cases 1-4) studied here. All four patients were identified as responders to CRT (Figures 1, 2).

**Table 1:** Patient characteristics and post-operative changes in the cardiac function and NYHA class following the CRT

Patient	Age	Gender	Etiology of HF	Rhythm	Medication			LVESV (pre/post) mL (% decrease)	LVEF (pre/post) % (net)	NYHA (pre/post)	Follow-up months
					RAS	BB	Diuretics				
Case 1	60s	Female	DCM	SR	ACEI	+	+	188/133 (27%)	26/31 (+5%)	III/II	7
Case 2	70s	Female	DCM	SR	MRB	+	+	129/109 (16%)	30/38 (+8%)	III/II	8
Case 3	60s	Male	Cardiac sarcoidosis	SR	ARB	+	+	286/194 (32%)	14/21 (+7%)	IV/II	9
Case 4	90s	Male	DCM	SSS	ARB	+	+	110/93 (15%)	31/36 (+5%)	IV/II	3

*ACEI*, angiotensin-converting enzyme inhibitor; *ARB*, angiotensin receptor blocker; *BB*, beta blocker; *DCM*, dilated cardiomyopathy; *HF*, heart failure; Lat, lateral wall; *LVEF*, left ventricular ejection fraction; *LVESV*, left ventricular end-systolic volume; *MRB*, mineral corticoid receptor blocker; *NYHA*, New York Heart Association; *OMI*, old myocardial infarction; pre/post, pre-operation/post-operation *SR*, sinus rhythm; *SSS*, sick sinus syndrome

## Discussion

This study demonstrated that the *FIVE STaR* method using a fusion imaging technique with CT coronary venography and myocardial perfusion SPECT successfully guided the operators in planning an effective CRT device implantation. In addition, the device treatment contributed to an improvement in the NYHA functional class and cardiac function such as in the LVESV and LVEF at least 3 months after the CRT implantation. The promising preliminary results indicated that the presented method could contribute not only to an appropriate selection of CRT responders, but also to a greater long-term efficacy of the CRT and subsequent improvement in the quality of life and outcomes in a cost-effective manner.

## Myocardial Viability and CRT

Besides the several non-negligible complications due to CRT,^15^,^16^ one of the important clinical issues to be resolved is how precisely can non-responders be distinguished from CRT responders who can obtain prognostic benefits from this device treatment. The CRT efficacy depends on a sufficient viable LV myocardial mass, which can improve the global LV function by resynchronization of any heterogeneous contraction abnormalities.^17^ The ATP-dependent mechanism behind the myocardial uptake of nuclear tracers such as thallium-201, Tc-99m-sestamibi, and Tc-99m-tetrofosmin enables the delineation and evaluation of the myocyte viability with a tracer activity of near 50% or more, facilitating the wide use of this imaging technique over the last few decades.^18^,^19^ Dobutamine-stress echo and perfusion CT imaging with a contrast agent are also promising but still not used widely for a myocardial viability assessment. Resting gated SPECT imaging has several advantages over them as follows. Three-dimensional assessment of the entire LV segments can be performed without any dead angle from the apical-to-basal and septal-to-lateral segments and with a high reproducibility (i.e., no significant inter- or intra-observer error). Use of an iodine contrast agent or stress protocol is not necessary for a myocardial viability assessment. Moreover, recently in addition to the quantitative assessment of the global and regional LV function, three-dimensional LV mechanical dyssynchrony can be evaluated using a phase analysis.^20^ Thus, resting gated myocardial perfusion SPECT imaging can provide the pictorial and quantitative information necessary for an appropriate identification of a CRT candidate with advanced HF and a myocardial region that can effectively respond to synchronized pacing, leading to the improvement in the LV function and subsequent outcomes.

## Coronary Venous Anatomy and CRT

Delayed imaging by coronary venography following conventional coronary angiography can be easily performed without any additional contrast agent or procedure in coronary CT imaging. The procedural planning in advance for a device implantation is important for operators to evaluate the appropriate access to an optimal pacing site and precise distance to viable myocardium among the coronary venous tree, in terms of the accessibility and effective pacing due to an appropriate pacing threshold. In addition, a precise understanding of the coronary vein anatomy prior to the implantation can contribute to the patient safety by reducing the operation time, volume of contrast agent (i.e., a risk of impairment of the kidney function), radiation exposure time, and risks for infections, bleeding, embolisms, or vein injuries^15^,^16^ related to the invasive procedures during the operation.

**Figure 3:**
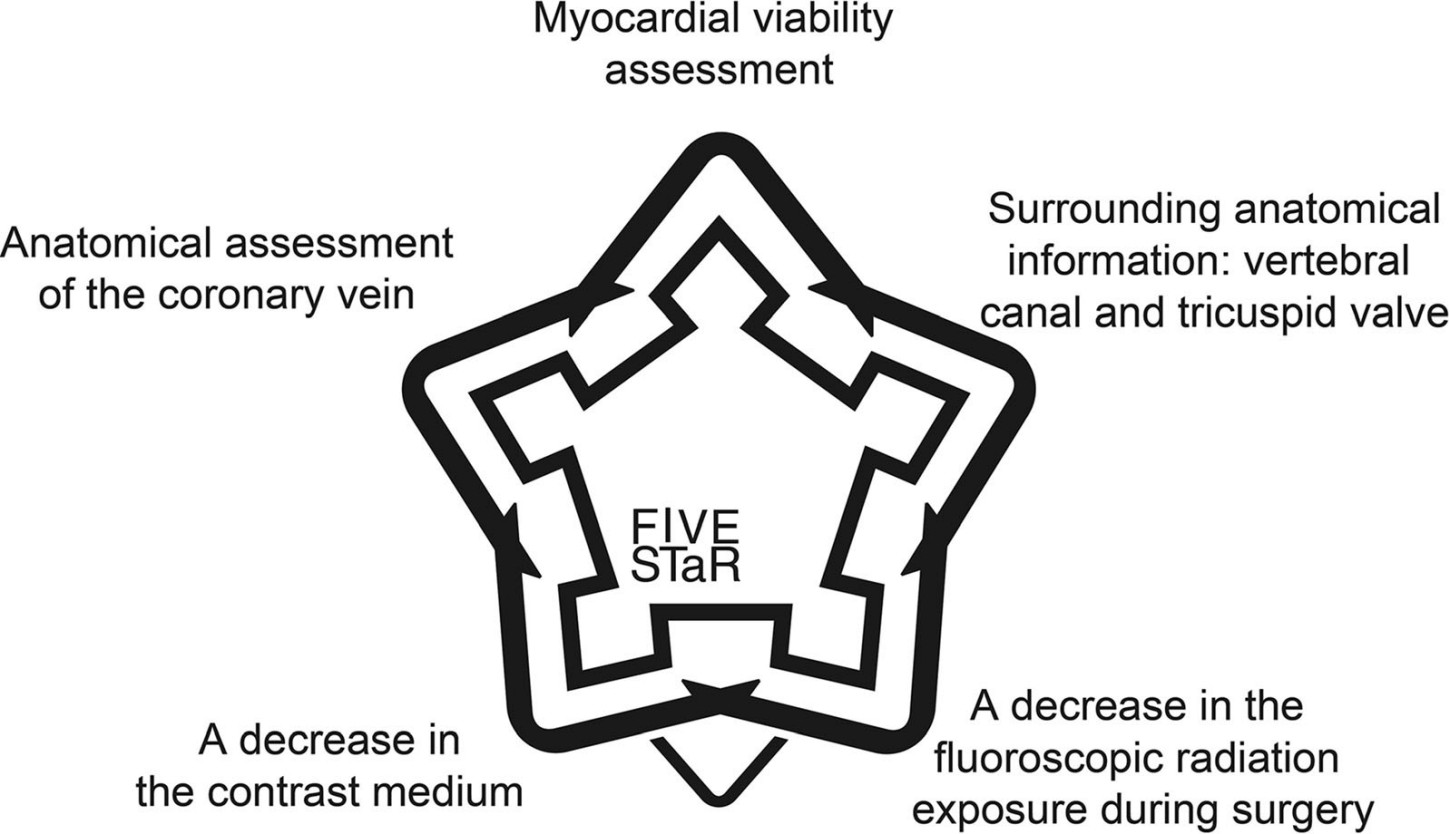
Summary of the five major advantages of the FIVE STaR method using CT coronary venography and myocardial perfusion imaging

More technically, it is helpful for operators to safely insert a guiding catheter and pacing lead into the targeted coronary vein by understanding the positional relationship between the surrounding anatomy and ostium of the great cardiac vein connected with the peripheral veins. CT venography can provide the information about the appropriate size, length, and shape of the guiding catheter and pacing lead, and the need for a subselection catheter. In contrast, coronary veins are not clearly visualized by conventional invasive coronary angiography and a subsequent venous appearance. Invasive retrograde coronary venography with an occlusion balloon also has limitations in clearly depicting the peripheral venous anatomy. Thus, the presented *FIVE STaR method* can improve the over-all patient safety, procedural success rate, and probably the CRT effectiveness by its various advantages (Figure 3).

## Three-Dimensional Fusion Imaging as a CRT Guide

Recent advances in CT technology^21^-^24^ such as dedicated software and improved spatial and temporal resolutions have enabled three-dimensional fusion imaging, clearly visualizing the correlation between the coronary venous anatomy and myocardial perfusion state as shown here. There have been reports of fusion of the fluoroscopy venogram and SPECT imaging.^25^,^26^ Instead of the fluoroscopic venogram, CT coronary venography using a 320-detector-row multi-slice CT was applied for the presented method. In this study, the cardiac CT and myocardial perfusion SPECT were performed separately. Although the spatial resolution between the cardiac CT and SPECT differed, this study demonstrated the sufficient accuracy of the presented method with an acceptable minimized fusion error, which may have reduced the clinical reliability. Nevertheless, misregistration may not be completely eliminated in each patient because of patient motion, uncontrollable heart rates during the CT imaging, and diffuse and/or large myocardial perfusion defects on SPECT imaging.

## Limitations

This small-sized study had several issues to be resolved in a future study. A large-scale, long-term study^9^,^27^ is strongly required to clarify the clinical efficacy of the *FIVE STaR* method in CRT candidates with advanced HF who meet the current CRT guidelines. A randomized or case-controlled study in combination with an outcome analysis will reveal the technical value of this method for CRT device implantations. The radiation exposure from scintigraphic and CT studies as well as during the device implantation has to be lowered at each stage of this method. It will be possible by improving the imaging protocol and using a gamma camera such as a semiconductor system to reduce the over-all radiation exposure and surgical time. The echocardiographic functional parameters (LVESV and LVEF) and NYHA functional class were used for monitoring the CRT response in this study. That was because of the lack of an absolute definition of a CRT response and because those indices seemed to be clinically acceptable. As shown by our previous study using gated SPECT imaging,^20^ a more direct parameter of the left ventricular dyssynchrony may be useful not only for evaluating the short-term efficacy but also for predicting the long-term outcomes. Finally, an improvement in the selection of appropriate CRT candidates (or non-CRT responders), viability assessment, and surgical procedure possibly would increase not only the clinical outcomes but also the cost-effectiveness of this device treatment, even though two modalities are required. The future advancements in multi-modality systems, such as a SPECT/(high-speed, multi-slice) CT system, may make the present method more time-saving and cost-effective. This method is also promising for improving the cost-effectiveness of CRT per se via the appropriate discrimination of non-responders to CRT and reducing any unnecessary CRT device implantations together with the additional information on myocardial ischemia, which can be indicated for other therapeutic strategies.

## New Knowledge Gained

We developed a novel approach, named the FIVE STaR method (Fusion Image using CT Venography and perfusion SPECT applied for cardiac Resynchronization therapy), for the optimization of CRT via a simultaneous assessment of the myocardial viability and an appropriate lead position using a fusion technique with CT coronary venography and myocardial perfusion imaging. The fusion image technique using myocardial perfusion imaging and CT coronary venography was clinically feasible and effective for CRT optimization in patients with advanced HF.

## Conclusion

The three-dimensional fusion imaging named the *FIVE STaR* method using myocardial perfusion imaging and CT coronary venography is clinically available and promising for facilitating the CRT optimization and providing an appropriate selection of CRT candidates and technical assistant for device implantation in patients with advanced HF.

## Electronic Supplementary Material

Below is the link to the electronic supplementary material.

Supplementary material 1 (PPTX 3179 kb)

## Abbreviations

CT: Computed tomography
CRT: Cardiac resynchronization therapy
CRTD: Cardiac resynchronization therapy and defibrillator
DCM: Dilated cardiomyopathy
FIVE STaR method: Fusion image using CT venography and perfusion SPECT applied for cardiac resynchronization therapy
HF: Heart failure
LVEF: Left ventricular ejection fraction
LVESV: Left ventricular end-systolic volume
MPI: Myocardial perfusion imaging
SPECT: Single photon emission computed tomography
