# Supplementary material for: A novel approach to the selection of an appropriate pacing position for optimal cardiac resynchronization therapy using CT coronary venography and myocardial perfusion imaging: FIVE STaR method (fusion image using CT coronary venography and perfusion SPECT applied for cardiac resynchronization therapy)
Source: J Nucl Cardiol. 2019 Aug 21;28(4):1438–45. doi: 10.1007/s12350-019-01856-z (PMC8421301; doi:10.1007/s12350-019-01856-z)
Supplement: Supplementary file 1 — Supplementary material 1 (PPTX 3179 kb) [file 12350_2019_1856_MOESM1_ESM.pptx]

## Slide 1
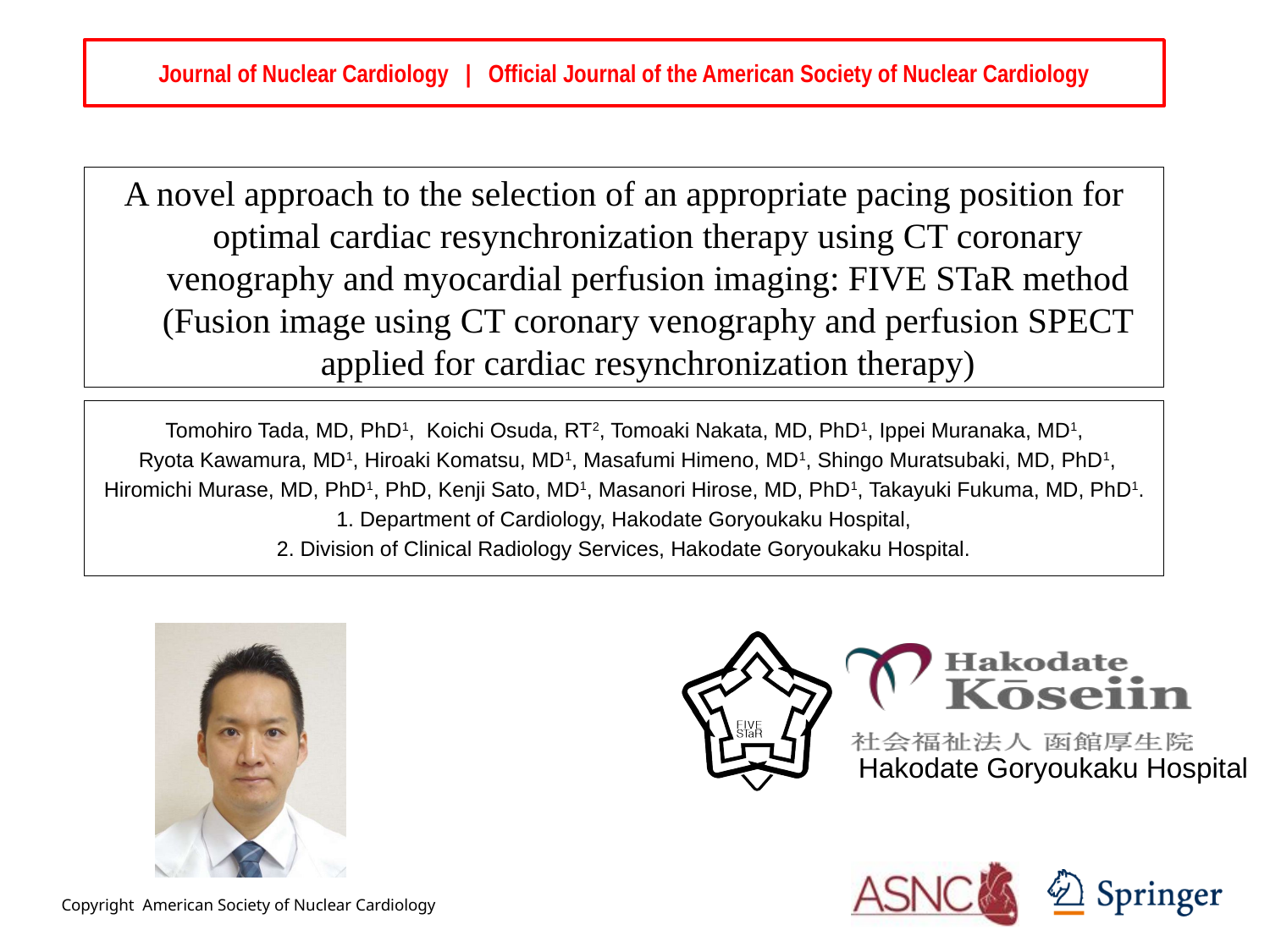

Journal of Nuclear Cardiology | Official Journal of the American Society of Nuclear Cardiology
# A novel approach to the selection of an appropriate pacing position for optimal cardiac resynchronization therapy using CT coronary venography and myocardial perfusion imaging: FIVE STaR method (Fusion image using CT coronary venography and perfusion SPECT applied for cardiac resynchronization therapy)
Tomohiro Tada, MD, PhD1, Koichi Osuda, RT2, Tomoaki Nakata, MD, PhD1, Ippei Muranaka, MD1,
 Ryota Kawamura, MD1, Hiroaki Komatsu, MD1, Masafumi Himeno, MD1, Shingo Muratsubaki, MD, PhD1,
 Hiromichi Murase, MD, PhD1, PhD, Kenji Sato, MD1, Masanori Hirose, MD, PhD1, Takayuki Fukuma, MD, PhD1.
1. Department of Cardiology, Hakodate Goryoukaku Hospital,
2. Division of Clinical Radiology Services, Hakodate Goryoukaku Hospital.
 Hakodate Goryoukaku Hospital
Copyright American Society of Nuclear Cardiology

## Slide 2
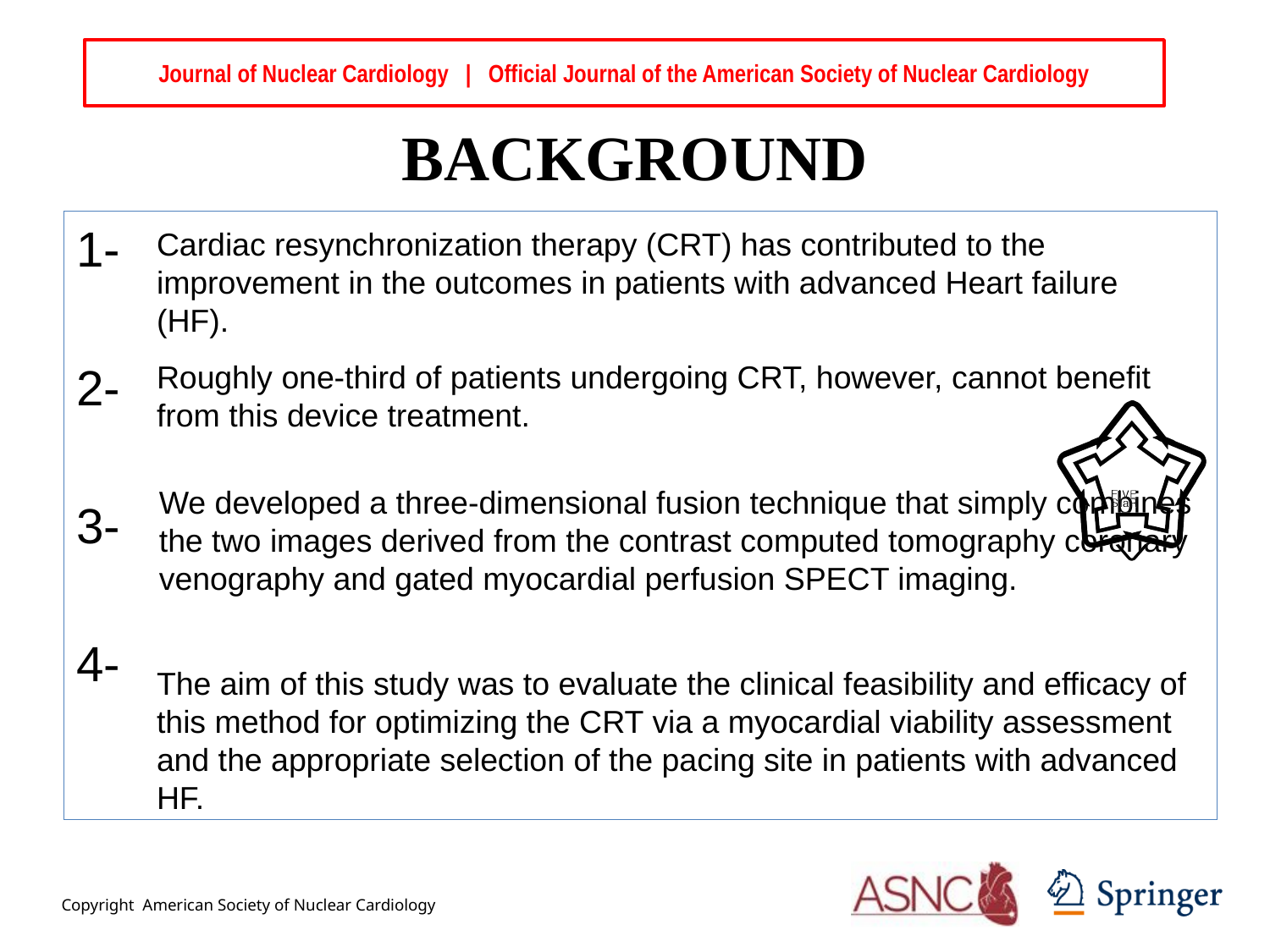

Journal of Nuclear Cardiology | Official Journal of the American Society of Nuclear Cardiology
# BACKGROUND
1-
2-
3-
4-
Cardiac resynchronization therapy (CRT) has contributed to the improvement in the outcomes in patients with advanced Heart failure (HF).
Roughly one-third of patients undergoing CRT, however, cannot benefit from this device treatment.
We developed a three-dimensional fusion technique that simply combines the two images derived from the contrast computed tomography coronary venography and gated myocardial perfusion SPECT imaging.
The aim of this study was to evaluate the clinical feasibility and efficacy of this method for optimizing the CRT via a myocardial viability assessment and the appropriate selection of the pacing site in patients with advanced HF.
Copyright American Society of Nuclear Cardiology

## Slide 3
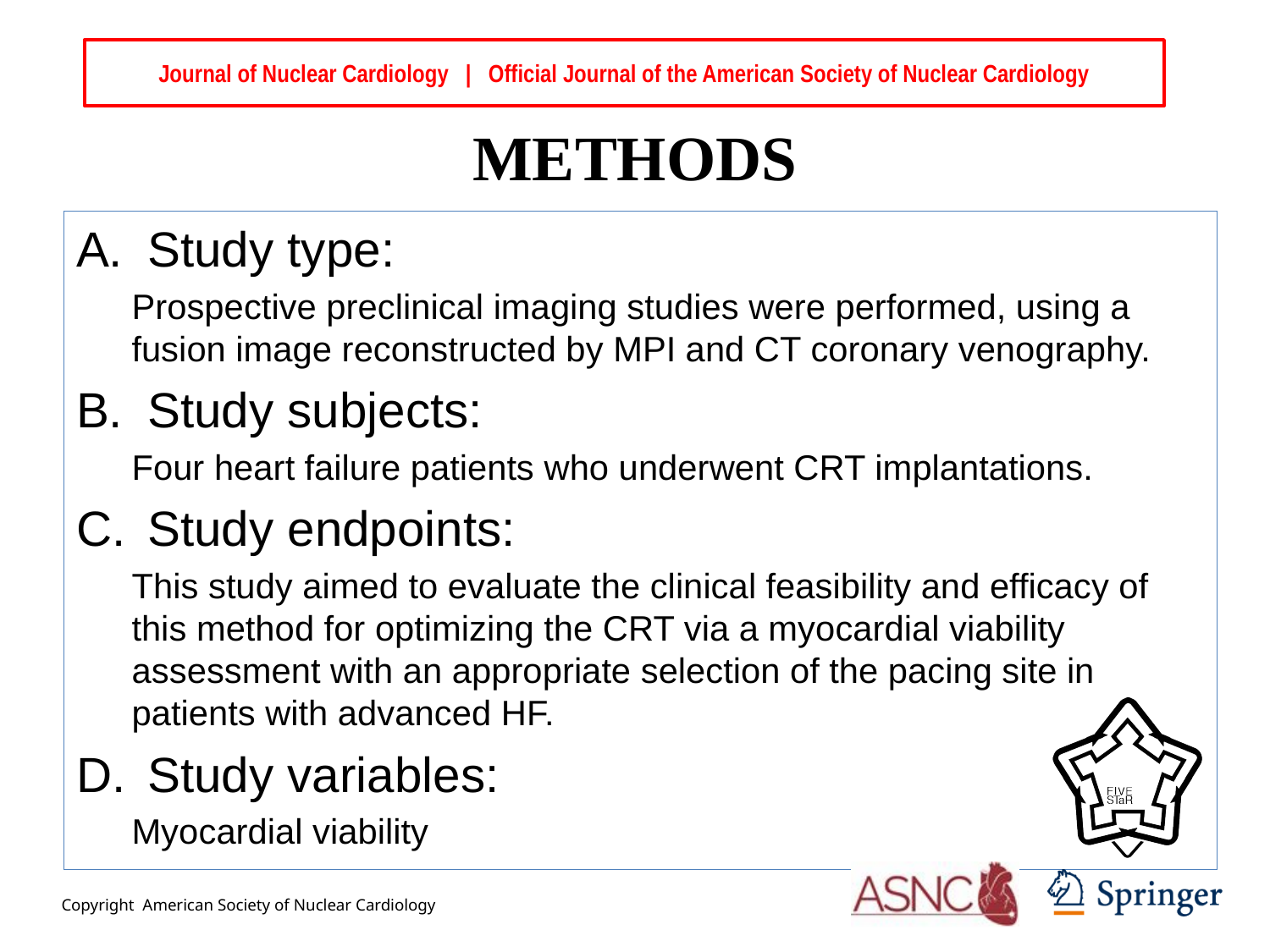

Journal of Nuclear Cardiology | Official Journal of the American Society of Nuclear Cardiology
# METHODS
Study type:
Prospective preclinical imaging studies were performed, using a fusion image reconstructed by MPI and CT coronary venography.
Study subjects:
Four heart failure patients who underwent CRT implantations.
Study endpoints:
This study aimed to evaluate the clinical feasibility and efficacy of this method for optimizing the CRT via a myocardial viability assessment with an appropriate selection of the pacing site in patients with advanced HF.
Study variables:
Myocardial viability
Primary end point(s):
Secondary end point(s):
Copyright American Society of Nuclear Cardiology

## Slide 4
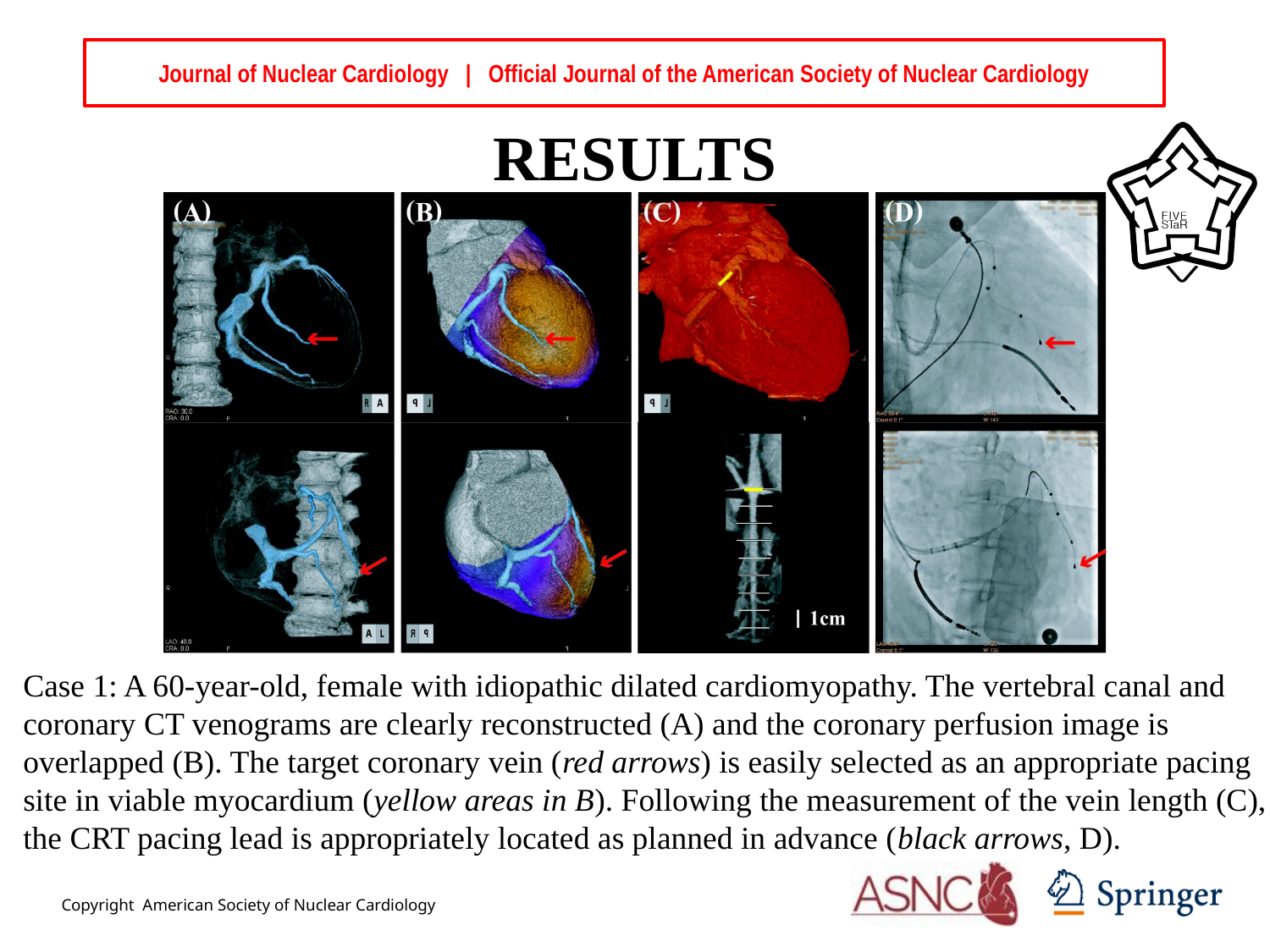

Journal of Nuclear Cardiology | Official Journal of the American Society of Nuclear Cardiology
# RESULTS
Case 1: A 60-year-old, female with idiopathic dilated cardiomyopathy. The vertebral canal and coronary CT venograms are clearly reconstructed (A) and the coronary perfusion image is overlapped (B). The target coronary vein (red arrows) is easily selected as an appropriate pacing site in viable myocardium (yellow areas in B). Following the measurement of the vein length (C), the CRT pacing lead is appropriately located as planned in advance (black arrows, D).
Copyright American Society of Nuclear Cardiology

## Slide 5
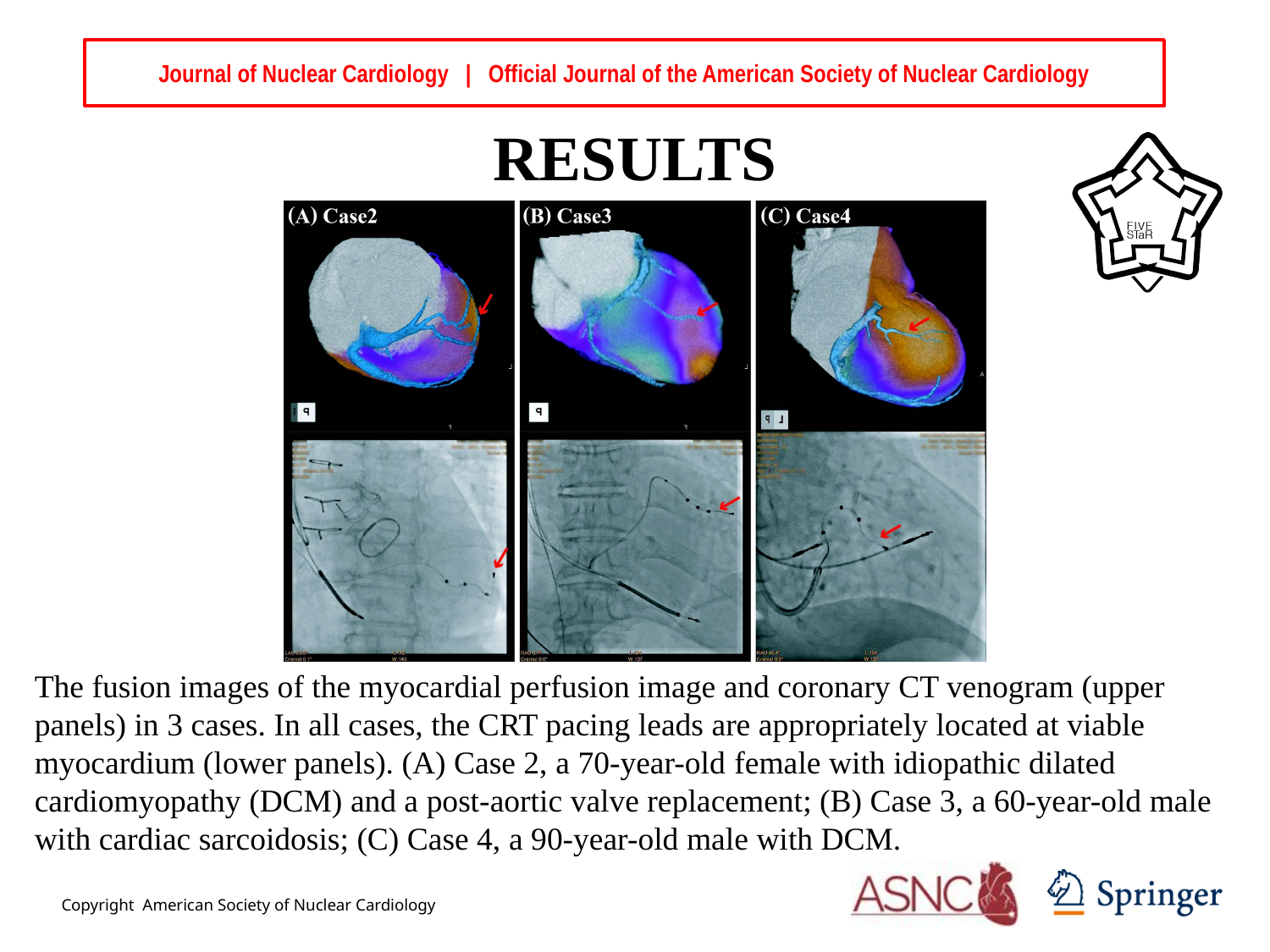

Journal of Nuclear Cardiology | Official Journal of the American Society of Nuclear Cardiology
# RESULTS
The fusion images of the myocardial perfusion image and coronary CT venogram (upper panels) in 3 cases. In all cases, the CRT pacing leads are appropriately located at viable myocardium (lower panels). (A) Case 2, a 70-year-old female with idiopathic dilated cardiomyopathy (DCM) and a post-aortic valve replacement; (B) Case 3, a 60-year-old male with cardiac sarcoidosis; (C) Case 4, a 90-year-old male with DCM.
Copyright American Society of Nuclear Cardiology

## Slide 6
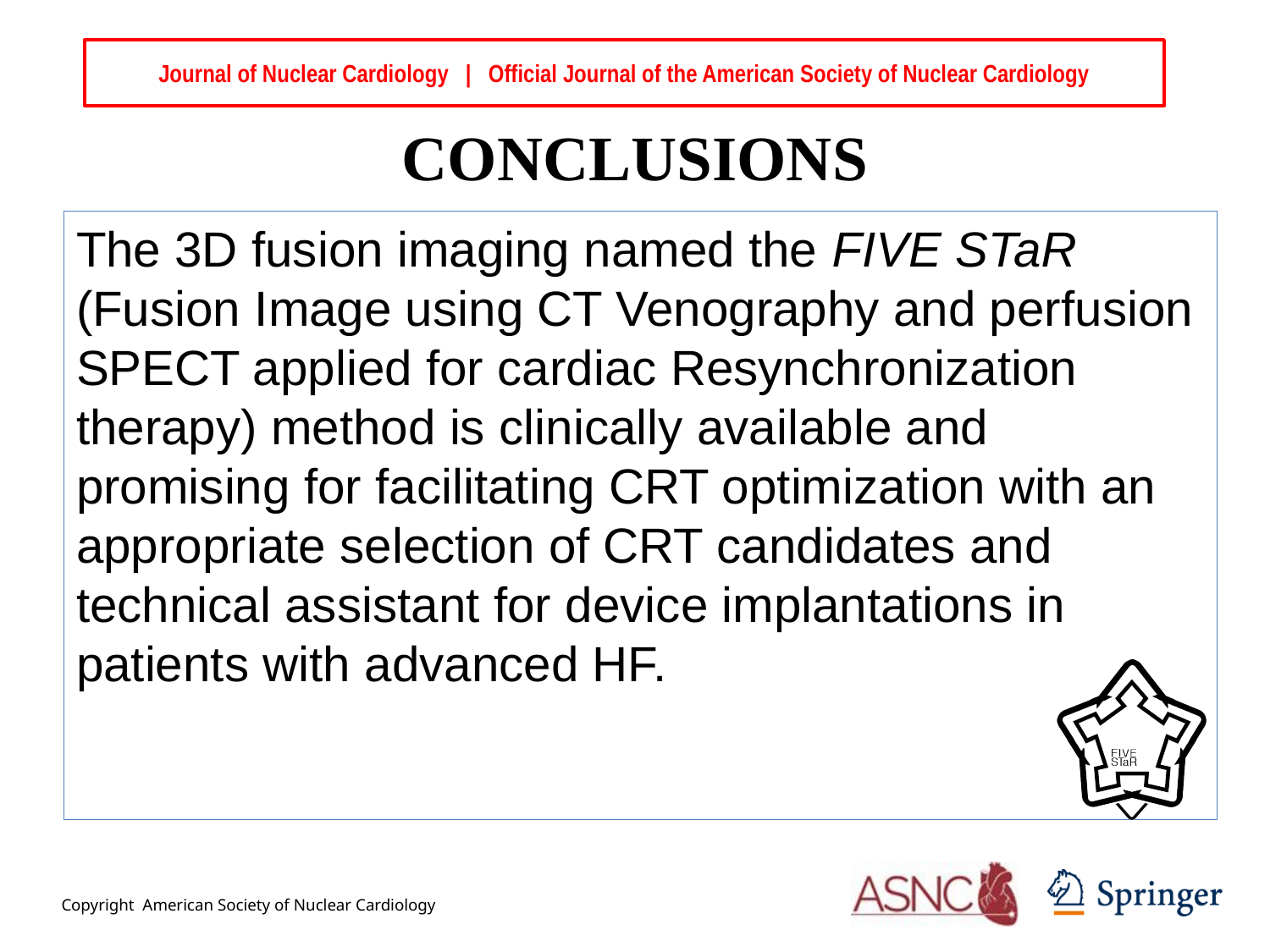

Journal of Nuclear Cardiology | Official Journal of the American Society of Nuclear Cardiology
# CONCLUSIONS
The 3D fusion imaging named the FIVE STaR (Fusion Image using CT Venography and perfusion SPECT applied for cardiac Resynchronization therapy) method is clinically available and promising for facilitating CRT optimization with an appropriate selection of CRT candidates and technical assistant for device implantations in patients with advanced HF.
Copyright American Society of Nuclear Cardiology
